# Autologous Transplantation of Oral Mucosal Epithelial Cell Sheets Cultured on an Amniotic Membrane Substrate for Intraoral Mucosal Defects

**DOI:** 10.1371/journal.pone.0125391

**Published:** 2015-04-27

**Authors:** Takeshi Amemiya, Takahiro Nakamura, Toshiro Yamamoto, Shigeru Kinoshita, Narisato Kanamura

**Affiliations:** 1 Department of Dental Medicine, Kyoto Prefectural University of Medicine, Graduate School of Medical Science, Kyoto, Japan; 2 Department of Ophthalmology, Kyoto Prefectural University of Medicine, Graduate School of Medical Science, Kyoto, Japan; 3 Research Center for Inflammation and Regenerative Medicine, Faculty of Life and Medical Sciences, Doshisha University, Kyoto, Japan; Instituto Butantan, BRAZIL

## Abstract

The human amniotic membrane (AM) is a thin intrauterine placental membrane that is highly biocompatible and possesses anti-inflammatory and anti-scarring properties. Using AM, we developed a novel method for cultivating oral mucosal epithelial cell sheets. We investigated the autologous transplantation of oral mucosal epithelial cells cultured on AM in patients undergoing oral surgeries. We obtained specimens of AM from women undergoing cesarean sections. This study included five patients without any history of a medical disorder who underwent autologous cultured oral epithelial transplantation following oral surgical procedures. Using oral mucosal biopsy specimens obtained from these patients, we cultured oral epithelial cells on an AM carrier. We transplanted the resultant cell sheets onto the oral mucosal defects. Patients were followed-up for at least 12 months after transplantation. After 2–3 weeks of being cultured on AM, epithelial cells were well differentiated and had stratified into five to seven layers. Immunohistochemistry revealed that the cultured cells expressed highly specific mucosal epithelial cell markers and basement membrane proteins. After the surgical procedures, no infection, bleeding, rejection, or sheet detachment occurred at the reconstructed sites, at which new oral mucous membranes were evident. No recurrence was observed in the long-term follow-up, and the postoperative course was excellent. Our results suggest that AM-cultured oral mucosal epithelial cell sheets represent a useful biomaterial and feasible method for oral mucosal reconstruction. However, our primary clinical study only evaluated their effects on a limited number of small oral mucosal defects.

## Introduction

Although oral mucosal defects created by tumor surgery or traumatic injury are augmented by mucosal transplantation, open wounds may become infected or develop contraction and secondary dysfunction. In such cases, autologous mucosal grafts provide transplant materials for postoperative mucosal defects in the oral cavity. However, autologous mucous membrane grafts leave defects at the donor site, and it may be difficult to harvest sufficient oral mucosa for reconstruction. The ideal reconstruction material is an autologous tissue, the harvesting of which involves minimally invasive procedures at the donor site. Tissue engineering studies have addressed these issues. Small masses of healthy oral mucosa have been cultured into mucosal epithelial cell sheets for use as biomaterials [1]. Cultured oral epithelial cell sheet transplants have the potential to respond to a wide range of membrane defects and represent an effective method with wide applications in surgical augmentation.

The human amniotic membrane (AM) is a tissue that has attracted considerable interest as a cell culture substrate that facilitates the proliferation, differentiation, and functional organization of various cell types *in vitro* [2–9]. It is a thin membrane of parenchymal tissue that covers the outermost surface of the placenta, and is generally discarded after parturition. It can be collected almost aseptically from the placenta with few technical problems or ethical concerns, and has unique characteristics, including anti-inflammatory and anti-infection properties [10, 11]. AM has been utilized as a tissue in various surgical therapies for the following purposes: to prevent adhesion/scarring after skin transplants or abdominal surgeries; as a wound dressing to accelerate the healing of burns; and in ocular surface reconstruction [12–16].

We investigated the possibility of reconstructing oral mucosal defects using an autologous cultured mucosal epithelium. We previously reported that oral mucosal epithelial cells grown on AM represented a useful biomaterial and feasible method for oral mucosal reconstruction [4, 6, 7]. In the present study, we attempted oral mucosal reconstruction using autologous AM-cultured oral mucosal epithelial sheets in five patients with oral mucosal defects after dental surgery. The use of AM in transplanted epithelial sheets following oral surgery has not yet been documented. This study represents the first step towards assessing the feasibility of transplanting autologous AM-cultured oral mucosal epithelial cell sheets in the oral cavity.

## Materials and Methods

### Ethics statement

All experimental procedures and clinical applications introduced here were approved by the Medicine Research Ethics Committee of Kyoto Prefectural University, Kyoto, Japan (R-29). We obtained prior written informed consent from all patients in accordance with the *Declaration of Helsinki* for research involving human subjects. The individual in this manuscript has given written informed consent (as outlined in PLOS consent form) to publish these case details.

### Subjects

We investigated the autologous transplantation of oral mucosal epithelial cells cultured on AM in patients undergoing oral surgeries. The study included five patients without any history of a medical disorder who underwent autologous cultured oral mucosal epithelial transplantation at our university hospital following oral surgical procedures and who were available for a follow-up exceeding 12 months. The patients comprised three men and two women who ranged in age from 36–75 years and had oral leukoplakia, pleomorphic adenoma, and mucous cysts (Table 1).

**Table 1:** Research patients who underwent amniotic membrane-cultured oral mucosal epithelial cell sheet transplantation.

Case	Age	Sex	Diagnosis	Site	Defect size (L×W×D)	Follow-up
1	74	Female	Oral leukoplakia	Buccal mucosa	17×12×2	24 months
2	45	Female	Pleomorphic adenoma	Upper lip	12×9×4	12 months
3	36	Male	Mucous cyst	Lower lip	18×16×2	18 months
4	42	Male	Mucous cyst	Lower lip	12×12×5	12 months
5	75	Male	Mucous cyst	Upper lip	13×12×5	12 months

L: length, W: width, D: depth: in mm.

### Preparation of the amniotic membrane

We obtained AM from women undergoing cesarean section. We collected tissue from AM for our clinical purposes. We sufficiently explained the proposed use to the women and obtained their informed consent. We washed the membranes under aseptic conditions with phosphate-buffered saline (PBS) containing 5 ml of 0.5% levofloxacin and stored them at −80°C in Dulbecco’s modified Eagle’s medium (GIBCO/Invitrogen, Carlsbad, CA, USA) and glycerol (1:1, v:v; Wako Pure Chemical Industries, Ltd., Osaka, Japan). AM was thawed immediately before use for the oral epithelial cell culture and washed three times with PBS. In the oral epithelial cell cultures, membranes were deprived of their amniotic epithelial cells by incubating with 0.02% EDTA (Nacalai Tesque, Inc., Kyoto, Japan) at 37°C for 2 h to loosen cellular adhesion, followed by gentle scraping with a cell scraper (Nalge Nunc International, Naperville, IL, USA) to remove the amniotic epithelial cells.

### Cultivation of oral mucosal epithelial cells on the amniotic membrane

We performed an oral mucosal epithelial cell culture according to a previously reported procedure with a few modifications [6]. Briefly, epithelial cells were co-cultured with mitomycin-C (MMC)-treated NIH/3T3 cells. Confluent 3T3 cells were incubated with PBS containing 4 μg/ml MMC (37°C, 2 h) in 5% CO_2_:95% atmospheric air to inhibit cell proliferation, rinsed with PBS to remove the MMC, trypsinized, and inoculated (2 × 10^4^ cells/cm^2^) into cell culture dishes.

We obtained oral mucosal biopsy specimens (approximately 3 mm in length) from each patient under local anesthesia 2–3 weeks before each surgical procedure for the oral epithelial cell sheet cultures on AM. Briefly, we carefully examined the oral mucosa and selected a biopsy site at the healthy mucogingival junction of the lower molar area or buccal mucosa. We injected 2% lidocaine hydrochloride containing epinephrine around the biopsy site to provide infiltration anesthesia. We made incisions of approximately 3 mm^2^ to create a biopsy site. We then obtain a sample containing submucosal connective tissue. We sutured the surgical wound with 3–0 surgical silk and removed all sutures 1 week later. We confirmed that the patients had negative results for various infections (including hepatitis B and C, syphilis, human immunodeficiency virus, Creutzfeldt-Jakob disease, and West Nile fever) on serum tests before performed biopsies.

We immersed the oral mucosal biopsy specimens in PBS containing 50 IU/ml penicillin-streptomycin for 10 min at room temperature (RT), incubated them with 1.2 IU dispase (37 °C, 1 h), and treated them with 0.05% trypsin-ethylenediaminetetraacetic acid (RT, 10 min) to separate the cells. Enzyme activity was eliminated by washing the specimens with culture medium. We centrifuged the cells twice at 1000 revolutions per minute for 5 min and suspended the cell pellet in culture medium at 1 × 10^5^ cells/cm^2^. We seeded the oral mucosal epithelial cells onto AM, left them to rest on cell culture plate inserts (Corning Inc., Corning, NY, USA), and then co-cultured them with MMC-treated 3T3 cells. The culture medium consisted of a 1:1 mixture of Dulbecco’s modified Eagle’s medium, Ham’s F-12 medium, and defined keratinocyte growth medium (ArBlast Co., Ltd., Kobe, Japan) supplemented with 5 μg/ml insulin, 10 ng/ml human recombinant epidermal growth factor, 50 IU/ml penicillin-streptomycin, and 5% autologous serum. We obtained autologous serum from all patients according to Nakamura *et al*. [17]. Blood samples (approximately 30 ml) were collected from the antecubital vein, centrifuged, and filtered under aseptic conditions, yielding approximately 10 ml of serum.

We submerged oral epithelial cells grown on AM in culture medium for approximately 2 weeks and then exposed them to air (i.e., air-lifting) for approximately 1 week. We incubated the cultures at 37°C in a 5% CO_2_:95% air incubator and changed the medium once daily.

### Immunohistochemistry

We performed immunohistochemical studies according to previously described methods [6]. We compared the cytoarchitectures and protein expression patterns of stained sections of oral mucosa and oral mucosal epithelial cells cultured on AM. Briefly, oral mucosal samples and AM-cultured oral mucosal epithelial cells were embedded in Tissue-Tek (Qiagen N.V., Venlo, Limburg, The Netherlands) compound, quick-frozen, and stored at −80°C. We prepared 8-μm-thick frozen sections from these stored specimens.

We fixed samples with acetone at 4°C for 10 min and then washed them in PBS at RT for 10 min. To block nonspecific binding, we incubated all specimens with 1% bovine serum albumin (Nacalai Tesque, Inc.) at RT for 20 min. We incubated the samples with the appropriate primary antibodies, presented in Table 2, at RT for 1 h, and rinsed them with PBS containing 0.15% Triton X-100. We subsequently incubated the samples at RT for 1 h with the appropriate secondary antibodies; namely fluorescein isothiocyanate-conjugated donkey anti-mouse immunoglobulin G antibodies (Molecular Probes, Inc., Eugene, OR, USA). We washed them again with PBS and mounted them under coverslips with an anti-fading medium (Vectashield; Vector Laboratories, Inc., Burlingame, CA, USA) containing propidium iodide as a nuclear counterstain for examination by confocal microscopy (Fluoview; Olympus Corporation, Tokyo, Japan).

**Table 2:** Primary antibodies and their sources.

Primary antibodies	Dilution	Sources
Mouse monoclonal cytokeratin 4	×200	Novocastra Laboratories, Newcastle-on-Tyne, UK
Mouse monoclonal cytokeratin 13	×200	Novocastra Laboratories, Newcastle-on-Tyne, UK
Mouse monoclonal cytokeratin 1	×40	Novocastra Laboratories, Newcastle-on-Tyne, UK
Mouse monoclonal cytokeratin 10	×50	Novocastra Laboratories, Newcastle-on-Tyne, UK
Mouse monoclonal integrin alpha 6	×200	Chemicon International, Inc., Temecula, CA, USA
Mouse monoclonal integrin beta 4	×500	Chemicon International, Inc., Temecula, CA, USA
Mouse monoclonal integrin alpha 3	×500	Chemicon International, Inc., Temecula, CA, USA
Mouse monoclonal integrin beta 1	×500	Chemicon International, Inc., Temecula, CA, USA
Mouse monoclonal laminin 5	×100	Chemicon International, Inc., Temecula, CA, USA
Mouse monoclonal laminin alpha 5 chain	×200	Chemicon International, Inc., Temecula, CA, USA
Mouse monoclonal collagen VII	×100	Chemicon International, Inc., Temecula, CA, USA

### Surgical procedures

We established an approximately 2-mm safety margin around the lesion, used a CO_2_ gas laser under local anesthesia to dissect the mucosa, and then detached the lesion. We transplanted the AM-cultured oral mucosal epithelial cell sheet onto the oral mucosal defect region after oral surgery and secured it with 7–0 nylon sutures (Prolene; Ethicon, Inc., Somerville, NJ, USA). We used fluorescein staining of the cultured oral epithelial sheets to confirm their quality. We also performed a tie-over dressing. We removed the threads on postoperative day 7.

## Results

### Cell morphology and keratin expression of oral epithelial cells cultured on the amniotic membrane

**Fig 1:**
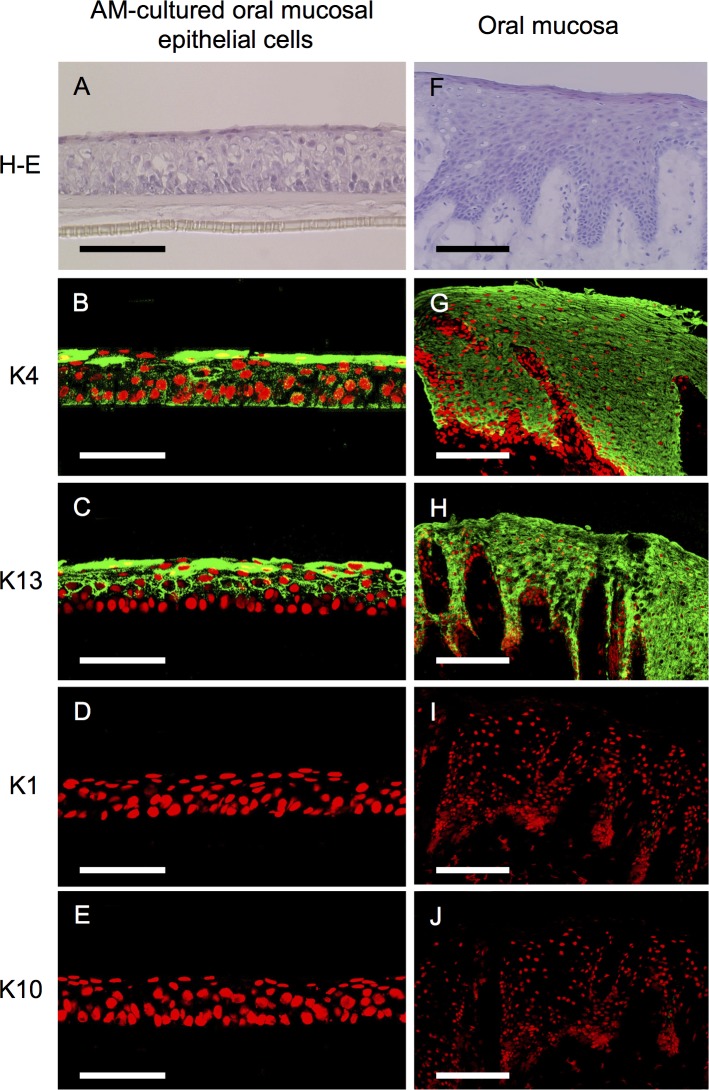
Morphology and keratin expression patterns of amniotic membrane-cultured oral mucosal epithelial cells and the oral mucosa. Light micrographs of amniotic membrane (AM)-cultured oral mucosal epithelial cells and the *in vivo* oral mucosa stained with hematoxylin and eosin as well as representative immunohistochemical staining results of AM-cultured oral mucosal epithelial cells and the *in vivo* oral mucosa. Culture oral mucosal epithelial cells grew well on AM, exhibiting five to seven differentiated, stratified layers with a measurable thickness (A). Keratins 4 and 13 were expressed by AM-cultured oral mucosal epithelial cells (B and C). These keratins were expressed in all epithelial layers of the oral mucosa (G and H). Conversely, keratins 1 and 10 were not expressed in any layer of AM-cultured oral mucosal epithelial cells (D and E) or oral epithelial cells (I and J). Nuclei are stained with propidium iodide (red). Scale bars: (A–E) 100 μm, (F–J) 200 μm.

After 2–3 weeks, the oral epithelial cells cultured on AM histomorphologically showed five to seven stratification layers of measurable thickness. The basal cells of the cultured epithelium were cuboidal, whereas the superficial cells were flattened (Fig 1A). Immunohistochemically, the oral epithelial cells stained positive for antibodies specific to the mucosal epithelium, e.g., keratins 4 and 13 (Fig 1B and 1C), which was consistent with our earlier findings [3]. Conversely, keratins 1 and 10 were not expressed in any layer (Fig 1D and 1E).

The oral mucosal epithelia revealed stratified cell layers (Fig 1F) and the expression of keratins 4 and 13 was evident throughout the oral mucosal epithelial layers (Fig 1G and 1H). In contrast, keratins 1 and 10 were not expressed at detectable levels in any layer (Fig 1I and 1J).

### Expression of basement membrane proteins in oral mucosal epithelial cells cultured on the amniotic membrane

**Fig 2:**
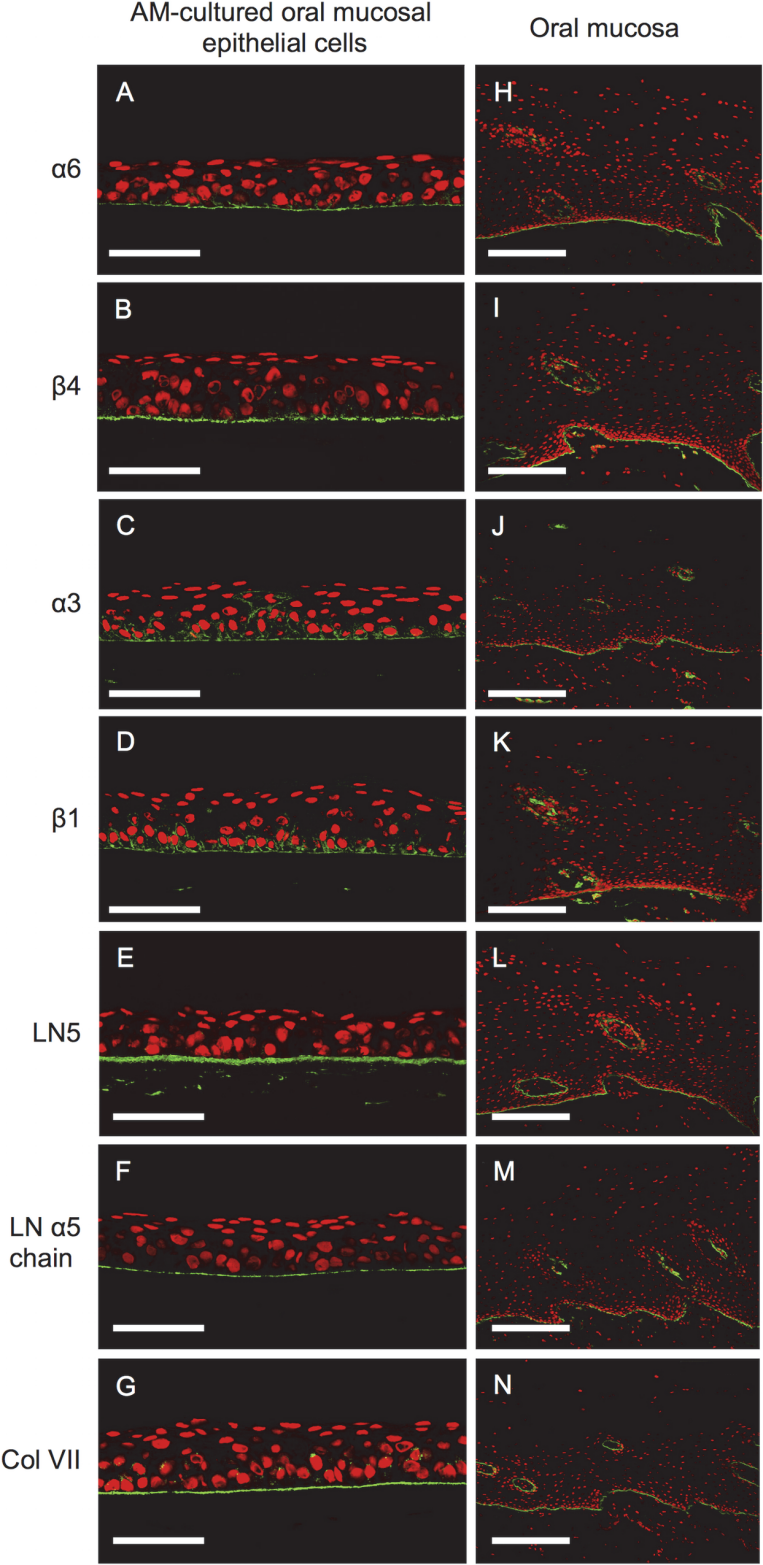
Expression of basement membrane proteins in amniotic membrane-cultured oral mucosal epithelial cells and the oral mucosa. Representative immunohistochemical results. Positive staining for integrins alpha-6 beta-4 and alpha-3 beta-1, laminin 5, the laminin alpha 5 chain, and collagen VII was evident on the basement membrane side of the cultured oral mucosal epithelial cell layer (A–G) and the basement membrane of the oral mucosa (H–N). Nuclei are stained with propidium iodide (red). Scale bars: (A–G) 100 μm, (H–N) 200 μm.

Immunohistochemical analyses of AM-cultured oral mucosal epithelial cells and oral mucosal epithelia demonstrated linearly positive staining for integrin alpha-6 beta-4 (Fig 2A, 2B, 2H and 2I), integrin alpha-3 beta-1 (Fig 2C, 2D, 2J and 2K), laminin 5 (Fig 2E and 2L), laminin alpha 5 chain (Fig 2F and 2M), and collagen VII (Fig 2G and 2N) on the basement membrane side. Expression patterns were similar between the cultured oral mucosal epithelial sheets and the *in vivo* oral mucosa.

### Transplanted amniotic membrane-cultured oral mucosal epithelial cell sheets

**Fig 3:**
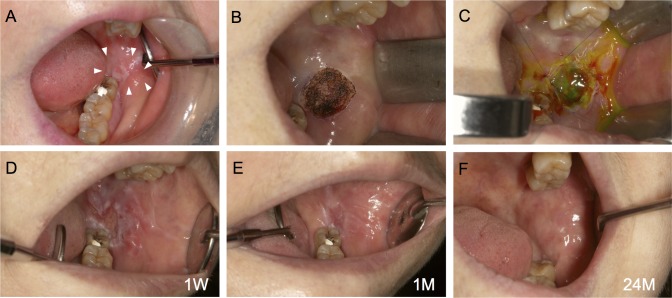
Autotransplantation procedure for an oral mucosal defect after surgery for oral leukoplakia: Case 1. A lesion measuring 15 × 10 × 2 mm was detected in the left buccal mucosa (A). The lesion was excised using a CO_2_ laser under local anesthesia (B). The amniotic membrane (AM)-cultured oral mucosal epithelial cell sheet was applied and sutured in place (C). One week after surgery, the transplanted sheets had adhered to the graft bed (D). Approximately 1 month after surgery, the mucosal defect had been replaced by the transplanted AM-cultured oral mucosal epithelial cell sheet (E). After 24 months, there was no postoperative recurrence (F).

**Fig 4:**
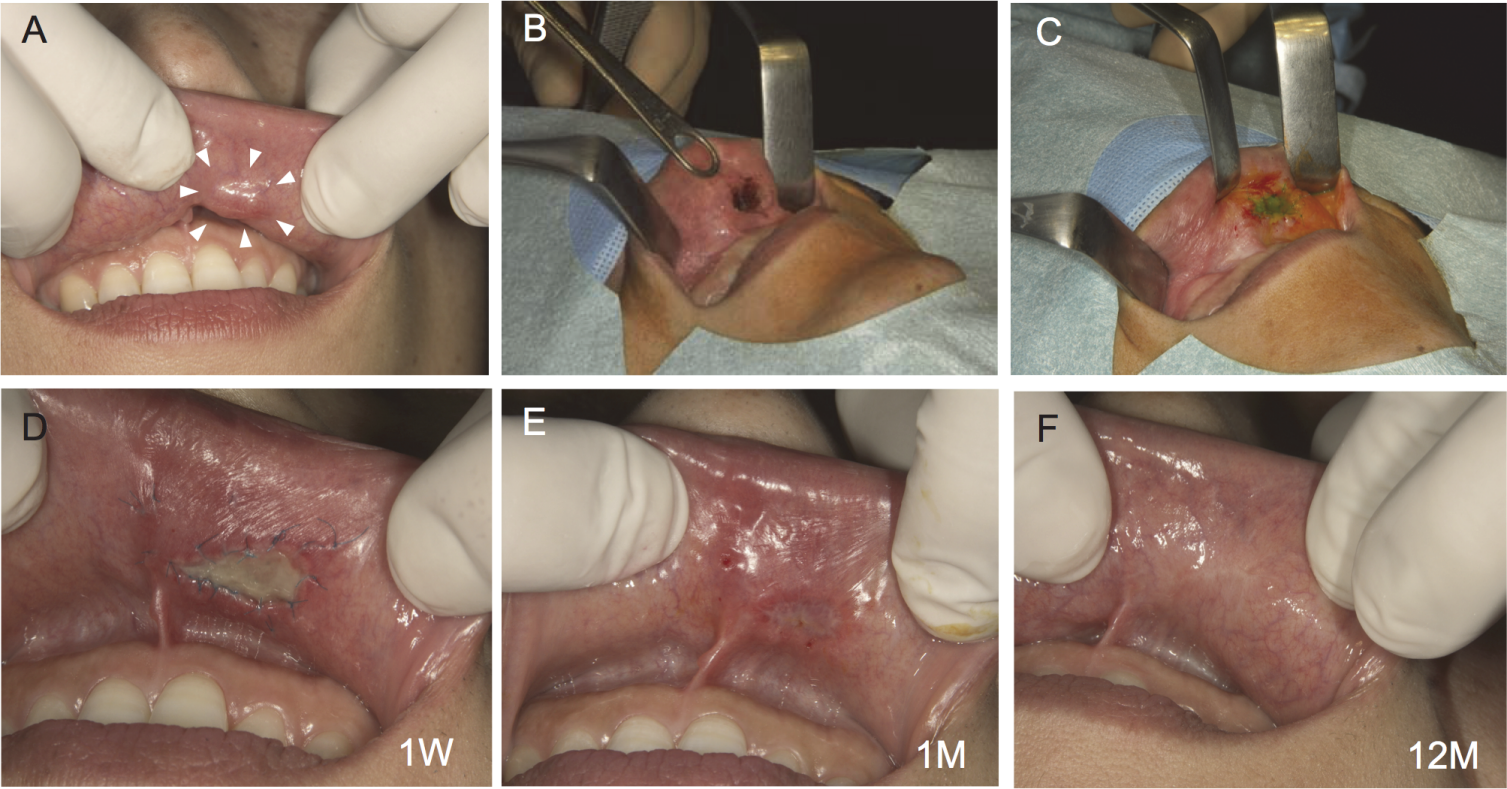
Autotransplantation procedure for an oral mucosal defect after surgery for pleomorphic adenoma: Case 2. A lesion measuring 10 × 7 × 4 mm was detected below the left mucosa of the upper lip (A). The lesion was excised to the depth of the subepithelial tissues using a CO_2_ laser under local anesthesia (B). An amniotic membrane (AM)-cultured oral mucosal epithelial cell sheet was applied to the oral mucosal defect and sutured in place (C). The sutures were removed 1 week after surgery. The transplanted site did not show infection, bleeding, or sheet detachment, and had adhered to the graft bed (D). Approximately 1 month after surgery, the mucosal defect had been replaced by the transplanted AM-cultured oral mucosal epithelial cell sheet (E). After 12 months, there was no postoperative recurrence (F).

**Fig 5:**
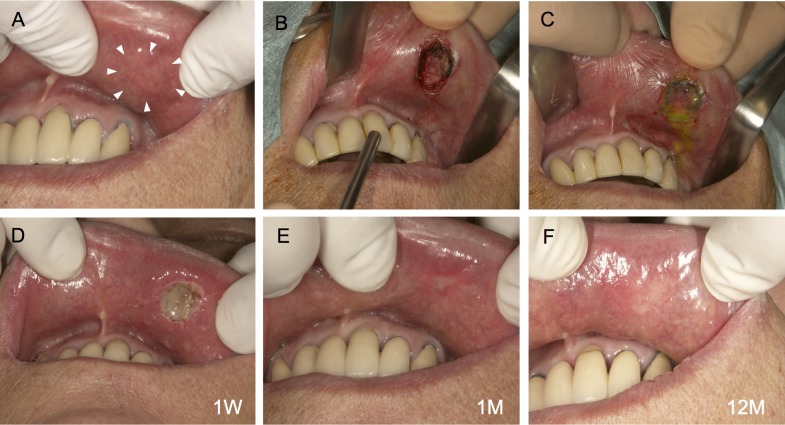
Autotransplantation procedure for an oral mucosal defect after surgery for a mucous cyst: Case 5. A lesion measuring 11 × 10 × 5 mm was detected below the left mucosa of the upper lip (A). The lesion was excised to the depth of the subepithelial tissues using a CO_2_ laser under local anesthesia (B). An amniotic membrane (AM)-cultured oral mucosal epithelial cell sheet was applied to the oral mucosal defect and sutured in place (C). The sutures were removed 1 week after surgery. The transplanted sheets had adhered to the graft bed (D). Approximately 1 month after surgery, the mucosal defect had been replaced with the transplanted AM-cultured oral mucosal epithelial cell sheet (E). After 12 months, there was no postoperative recurrence (F).

Autologous AM-cultured oral mucosal epithelial cell sheets were successfully generated for all five patients. Clinically, AM-cultured oral mucosal epithelial cell sheets had sufficient strength to be handled. In all cases, we observed that the transplanted sheets adhered to the mucosal defects (Figs 3A–3C, 4A–4C and 5A–5C). One week after surgery, no infection, bleeding, or sheet detachment was evident at the transplanted sites. The transplanted cell sheets were visible to the naked eye as adherent to the graft beds, and were distinguishable from the surrounding adjacent mucosa (Figs 3D, 4D and 5D).

At 1 month, the mucosal defects were well-epithelialized and had been replaced by transplanted oral epithelial sheets. They were macroscopically indistinguishable from the surrounding mucosa (Figs 3E, 4E and 5E). We did not detect recurrence macroscopically in the long-term follow-up, which exceeded 12 months, and all patients had an excellent postoperative course (Figs 3F, 4F and 5F). Cases 1 and 2 were reported previously (Figs 3 and 4) [4, 7], and the long-term follow-up of these cases was performed in this study.

## Discussion

The acquisition of transplantable cultured structures requires a combination of stem cells, an adequate extracellular matrix, and growth factors. Cultured oral epithelial cell sheets have been developed for mucosal defects; however, these epithelial sheets lack substrates and are fragile and difficult to handle for grafting [1]. The important element in oral grafting is a substrate that supports the oral epithelial cells. Selecting a substrate with biocompatibility, stability, and mechanical properties is important for tissue engineering. We used AM as a key substrate for the growth of oral mucosal epithelial cells and performed preliminary clinical studies on AM-cultured oral mucosal epithelial cell sheets [17] for intraoral mucosal defects. To the best of our knowledge, this is the first study to have successfully transplanted these cells into the oral cavity.

AM is a thin placental membrane that retains amniotic fluid and is easily attainable. Morphologically, AM consists of a monostratal amniotic epithelium on a basement membrane of a specific parenchymal thickness. Immunohistochemically, collagens III and IV and laminin are expressed in the amniotic basement membrane; collagens are also expressed in the parenchyma [18–20]. In addition, AM contains many growth factors, including epidermal growth factor, keratinocyte growth factor, hepatocyte growth factor, and basic fibroblast growth factor [21], and exhibits anti-inflammatory [22] and antibacterial properties [23]. The biologically active peptides in AM have significant roles in epithelial cell proliferation. Our results demonstrated that AM is suitable for culturing oral mucosal epithelial cells, suggesting that it is a useful tissue for normal cell proliferation and differentiation. Although little is known about its mechanism of action, it can be expected to elicit a relatively mild immune response. Several advantages of the clinical use of AM as a tissue have been reported in the field of surgical procedures. It has also been used in the fields of reconstructive and dental surgery, dermatology, and ophthalmology, and significant evidence of its success has been reported [13, 14, 24]. In addition, AM can be used not only as a transplant material but also as a substrate for cell culture [1–9, 15, 17, 24, 25].

An important element in oral mucosal reconstruction is the scaffold that supports the cells. In our previous study, we focused on AM as a scaffold to facilitate the growth of oral epithelial cells and successfully transplanted AM-cultured autologous mucosal epithelial cells onto intraoral mucosal defects in animals [6]. No rejection or inflammatory reactions occurred, underscoring the potential use of AM as a graft material. Nakamura *et al*. [17, 26] produced AM-cultured oral epithelial cell sheets as an extracellular matrix. They obtained good clinical results using these sheets to treat severe intractable corneal disorders. We previously confirmed the detachment/removal of amniotic epithelial cells was using electron microscopy [27]. We did not quantify DNA in AM after the removal of epithelial cells (i.e., denuded AM); however, we succeeded in clinically applying denuded AM and autologous oral mucosal epithelial cells to ocular surface reconstruction in patients with intractable corneal/conjunctival diseases [26]. No serious adverse events developed after transplantation over a long-term follow-up [17].

To the best of our knowledge, no previous studies have documented the use of AM as a scaffold for human oral mucosal epithelial cell sheets; therefore, it was unclear whether these cells had the ability to proliferate efficiently or differentiate appropriately on AM. Furthermore, it had not been determined whether oral mucosal epithelial cells grown on AM were an effective biomaterial for reconstructing oral mucosal defects in clinical settings. Collection of the oral mucosa (biopsy) is associated with the risk of developing various postoperative complications, including pain, swelling, bleeding, and functional disorders. Thus, we explained the possible development of these complications to the patients undergoing biopsy and obtained their consent. We also avoided the risk of postoperative complications and cicatrization of the donor site by reducing the size of the biopsy specimen as much as possible (approximately 3 mm in length). In addition, we strictly followed the course by administering oral antibiotics to prevent infection and anti-inflammatory analgesics to reduce pain and confirmed the absence of abnormal findings such as secondary infection. No serious postoperative complication developed and patient satisfaction was high. We previously compared cultures of oral mucosal epithelial cells between media containing autologous serum and general fetal bovine serum [25]. Cell growth within autologous serum and fetal bovine serum was equivalent; oral mucosal epithelial cells cultured with autologous serum on AM expressed mucosa-specific keratins 4 and 13, and the basement membrane (integrin alpha-6, beta-4, and beta-1; collagen VII; and laminin 5) and hemidesmosomes were present.

We immunohistochemically analyzed the oral epithelial cells cultured on AM to examine the ability of this scaffold material to maintain the characteristics of the oral mucosa, namely, cell proliferation and basal cell attachment to the underlying substrate. The cultivation of oral mucosal epithelia on AM at the air-liquid interface facilitated the construction of multilayered sheets of an epithelium that resembled the native epithelium and showed signs of differentiation such as basement membrane formation and cytokeratin expression [15, 28, 29]. Human oral epithelial cells cultured for 3 weeks on AM manifested five to seven differentiated, stratified layers; however, stratification was insufficient and complete cornification was rarely observed. We chose a 2–3-week culture period in this study based on our earlier findings that cell growth decreased in longer cultures [3, 6, 7, 15]. Immunohistochemically, keratins 4 and 13 are highly specific mucosal epithelial cell markers [30–33], whereas keratins 1 and 10 are cornified epidermis markers [31, 33, 34]. Our immunohistochemical results indicated that AM-cultured human oral epithelial sheets expressed keratins 4 and 13, but not 1 or 10, which was consistent with the findings of our earlier animal experiment [6]. This showed that AM-cultured oral mucosal epithelial cells maintained many of the phenotypic characteristics of oral mucosal epithelia *in vivo*.

An ideal engineered oral mucosa closely resembling a normal oral mucosa should consist of a continuous basement membrane that separates the lamina propria and epithelium [35], with a stratified squamous epithelium on the basement membrane [1]. Integrins alpha-3 beta-1 and alpha-6 beta-4, which are specific receptors for laminin 5, are widely distributed in the basement membranes of epithelial tissues, and laminin 5 has two functional domains capable of binding these integrins and collagen VII on basal cells to form anchoring fibrils [36, 37]. Collagen VII and the laminin alpha 5 chain are major components of basement membranes, and collagen VII localizes exclusively to the basement membrane zone. Integrin alpha-6 beta-4 is a specific component of hemidesmosomes [38].

Since integrin alpha-6 beta-4 and alpha-3 beta-1, laminin 5, the laminin alpha 5 chain, and collagen VII were expressed in the basal layer of AM-cultured oral mucosal epithelial cell sheets, these results indicated that AM-cultured oral mucosal epithelial cell sheets maintained the properties of the mucous membrane. The reaction products of these proteins were confined to basal epithelial cells adherent to AM with hemidesmosome attachments and produced the basement membrane of the oral mucosal epithelia in culture.

Clinically, a key point for successfully cultivating oral epithelial sheets is understanding how basal cells attach to the underlying AM, and these findings encouraged us to perform the transplantation of AM-cultured oral epithelial cells. Therefore, the transplantation of AM-cultured oral mucosal epithelial cells may be considered advantageous because rapid epithelialization occurs following transplantation; this makes the transplanted site very stable and avoids postoperative infections, both of which are important factors for the successful clinical application of this procedure. In the present study, we clinically applied AM-cultured oral mucosal epithelial cell sheets and encountered no problems in the strength of the cell sheets or their applicability during surgery. No complications such as immunological rejection or infection occurred in any patient.

Izumi *et al*. [39] reported that cultured oral mucosal epithelial grafts enhanced the maturation of the underlying submucosal layer and were associated with rapid epithelial coverage in intraoral applications. In our study, we could not obtain histological samples from the transplanted areas. Macroscopically, the transplanted sheets were visible to the naked eye as adherent to the graft beds and were distinguishable from the adjacent mucosa at 1 week. After 1 month, the mucosal defects were well-epithelialized and had been replaced by the transplanted oral epithelial sheets. The transplanted oral epithelial sheets became macroscopically indistinguishable from the surrounding mucosa over time, suggesting that AM-cultured oral mucosal epithelial cell sheets were successfully engrafted onto the oral mucosal defects.

In conclusion, we succeeded in growing oral epithelial cells on AM and performing the autologous transplantation of these cells onto intraoral mucosal defects. This primary clinical study evaluated a limited number of small oral mucosal defects. In future studies, we aim to examine the utility of AM-cultured oral mucosal epithelial sheets on lesions of an extensive size, depth, and in different regions in a larger number of patients.
